# Anesthetic management of a patient with methamphetamine-associated pulmonary arterial hypertension undergoing laparoscopic cholecystectomy

**DOI:** 10.1186/s40981-023-00601-3

**Published:** 2023-02-20

**Authors:** Kensuke Oshita, Shin Tokuyama, Shosaburo Jotaki, Michiko Yokomizo, Teruyuki Hiraki

**Affiliations:** Department of Anesthesiology, Kurume University School of Medicine, 67 Asahi-Machi, Kurume, Fukuoka 830-0011 Japan

**Keywords:** Pulmonary arterial hypertension, Methamphetamine, Right ventricular dysfunction, Peritoneal insufflation, Pulmonary artery catheter

## Abstract

**Background:**

Methamphetamine abuse is a serious public health concern and causes various life-threatening disorders including pulmonary arterial hypertension (PAH). Herein, we present the first case report describing the anesthetic management of a patient with methamphetamine-associated PAH (M-A PAH) undergoing laparoscopic cholecystectomy.

**Case presentation:**

A 34-year-old female with M-A PAH suffered from deterioration of right ventricular (RV) heart failure due to recurrent cholecystitis and was scheduled for laparoscopic cholecystectomy. Preoperative assessment of PA pressure showed 82/32 (mean, 50) mmHg, and transthoracic echocardiology revealed a slight reduction of RV function. General anesthesia was induced and maintained by thiopental, remifentanil, sevoflurane, and rocuronium. PA pressure gradually increased after peritoneal insufflation; therefore, we administered dobutamine and nitroglycerin to decrease pulmonary vascular resistance (PVR). The patient emerged from anesthesia smoothly.

**Conclusions:**

Avoiding increased PVR by appropriate anesthesia and medical hemodynamic support is an important consideration for patients with M-A PAH.

## Background

Methamphetamine is a highly addictive drug, which releases neurotransmitters, such as serotonin, norepinephrine, and dopamine, in the brain, resulting in psychokinetic change [1]. Chronic methamphetamine abuse affects the systemic organ system, leading to psychosis, cerebral stroke, and cardiovascular complications, especially pulmonary arterial hypertension (PAH) [2, 3]. To the best of our knowledge, there are no reports describing the anesthetic management of patients with methamphetamine-associated (M-A) PAH because the incidence of this disease is very low. Herein, we present the first case report describing the anesthetic management of a patient with M-A PAH undergoing laparoscopic cholecystectomy.

## Case presentation

A 34-year-old woman (height 155 cm, weight 89 kg) suffered from recurrent cholecystitis and was scheduled to undergo laparoscopic cholecystectomy under general anesthesia. She had PAH that was induced by abuse of various drugs, particularly methamphetamine. She had long-term, repeated exposure to methamphetamine and toluene from 17 to 28 years old. When she was 31 years old, she developed whole-body edema and dyspnea and was diagnosed with right ventricular (RV) heart failure due to M-A PAH based on sufficient exposure to methamphetamine and exclusion of other common cases of PAH. Pulmonary vascular resistance (PVR) decreased after the administration of epoprostenol (prostaglandin I2); however, alveolar hemorrhage induced by high pulmonary perfusion occurred. She developed hemoptysis and respiratory failure, which required mechanical ventilation. At that point, we observed tolerance to the sedative effect of propofol.

Preoperative assessment of pulmonary arterial catheterization revealed that pulmonary arterial pressure (PAP) and PVR were 82/32 (mean, 50) mmHg and 460 dynes･s/cm^5^, respectively, with a normal cardiac index of 3.6 L/min/m^2^. She was receiving multiple PAH-specific medicines, including macitentan 10 mg, tadalafil 40 mg, selexipag 3.2 mg, and sildenafil citrate 60 mg, and several benzodiazepines and antidepressants for the treatment of sleep impairment. Her activated partial thromboplastin clotting time was 47.1 s. The cardiologist and anesthesiologist assessed that her intraoperative risk was very high in accordance with previous reports showing higher mean PAP as a risk factor for increased mortality [4–6]. We obtained informed consent for cardiorespiratory resuscitation and extracorporeal membrane oxygenation if critical complications occurred.

**Fig. 1:**
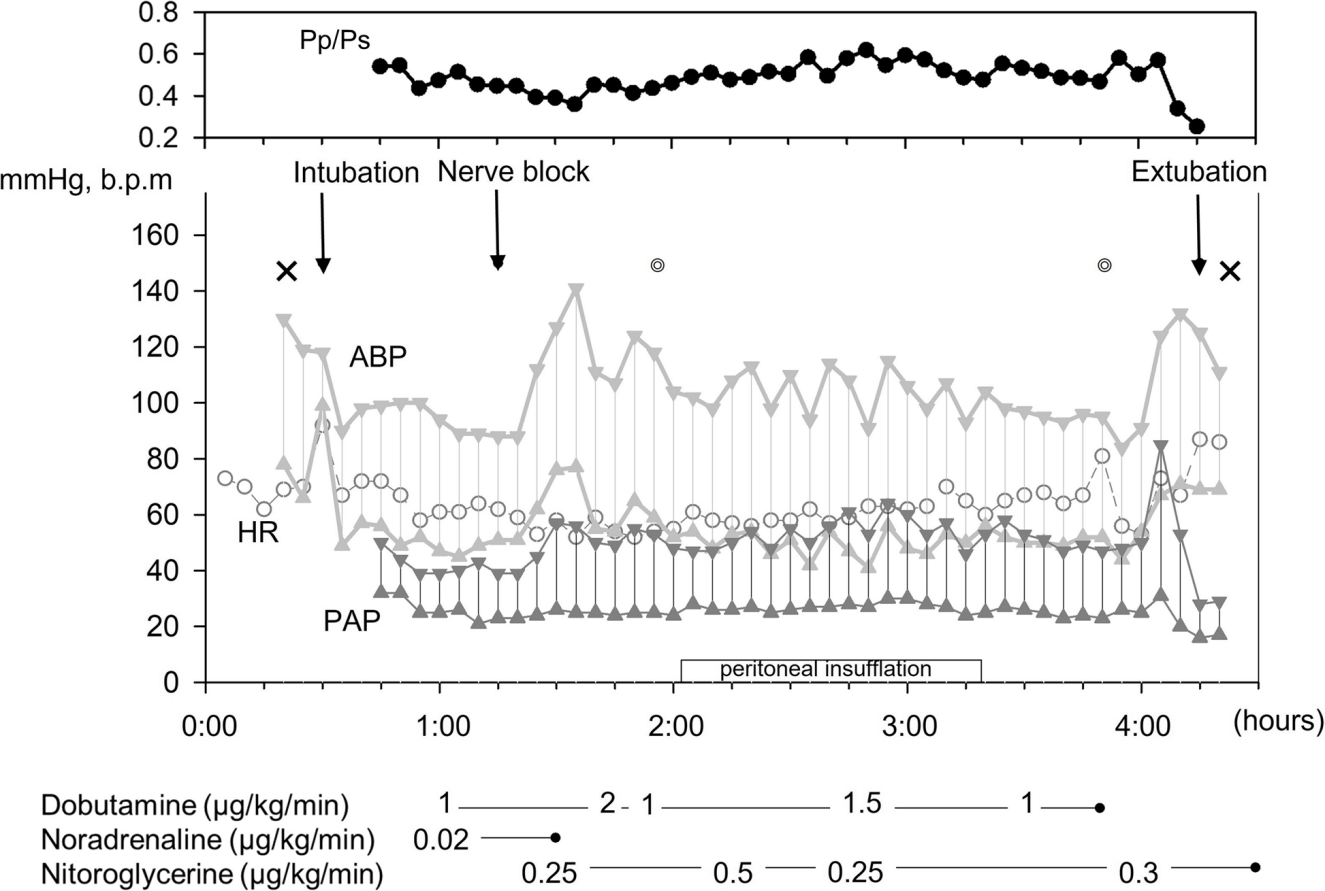
Anesthesia record. The ratio of mean pulmonary artery pressure to mean artery pressure (Pp/Ps) was increased from 0.36 to 0.58 after peritoneal insufflation, requiring an increase in the infusion rate of nitroglycerine. X: start and end of anesthesia; ◎: start and end of the operation. ABP, arterial blood pressure; HR, heart rate; PAP, pulmonary arterial pressure; Pp/Ps, the ratio of mean pulmonary artery pressure to mean artery pressure

On arrival in the operating room, we used the standard monitors, including a pulse oximeter, electrocardiograph, capnometer, and bispectral index (BIS) monitor, and monitored the artery blood pressure via a radial artery. In addition, we placed a pulmonary arterial catheter and performed transesophageal echocardiography (TEE) after induction of anesthesia and intubation. Anesthesia was induced with thiopental, rocuronium, and remifentanil and maintained by sevoflurane, remifentanil, fentanyl, and rocuronium. After intubation, we performed a transverse abdominal plane block for postoperative pain control. The ventilation settings were a tidal volume of 5 ml/kg (450 ml), positive end-expiratory pressure of 5 cmH_2_O, and ventilation rate of 12/min. The BIS value was between 47 and 60 during the operation. As shown in Fig. 1, the mean PAP and the ratio of mean PAP to mean artery pressure were gradually elevated from 28 to 40 mmHg and 0.36 to 0.58, respectively, after peritoneal insufflation was initiated at 8 mmHg. We regulated the dosage of dobutamine and nitroglycerin, and the target range of endo-tidal CO_2_ was set around 35 mmHg to decrease PVR. Intraoperative TEE demonstrated RV dilation without significant change after peritoneal insufflation. Cholecystectomy was uneventfully performed, followed by the replacement of the tracheal tube with the i-gel (Intersurgical Ltd., UK) and discontinuation of anesthesia.

When all procedures had been performed, anesthesia was discontinued. The patient was extubated after confirming smooth emergence. The patient was transferred to an intensive care unit, and the mean PAP was around 40 mmHg during the intensive care unit stay. Three days after the operation, she was discharged without complication.

## Discussion

We experienced the anesthetic management of the case with M-A PAH who underwent laparoscopic cholecystectomy. Although the 2018 World Symposium on Pulmonary Hypertension categorized methamphetamine use as a definite cause of PAH [3], anesthetic management for patients with M-A PAH is not well established because of the rarity of the disease. The present case report firstly describes the anesthetic management for an M-A PAH patient, based on preventing increased PVR during anesthesia.

Evidence supporting the association between methamphetamine and PAH has been accumulated. A previous study demonstrated that rates of prior methamphetamine use in patients with idiopathic PAH were 8–10 times higher compared with other forms of PAH [7]; thus, this suggests that there are many patients with M-A PAH that have not been diagnosed with it. Although the mechanism of PAH induced by methamphetamine abuse is not fully understood, the proliferation of pulmonary vascular smooth muscle cells by serotonin released by methamphetamine would lead to the thickening of the pulmonary vessel walls [8]. However, not every patient with a history of methamphetamine use develops PAH, suggesting that susceptibility to methamphetamine-related vascular injury by genetic potential may be involved in M-A PAH. In a previous study, bone morphogenetic protein receptor type II gene mutations were reported in 30% of patients with PAH [9]. So far, whether M-A PAH is reversible with cessation of methamphetamine and how much methamphetamine use develops a risk of M-A PAH remain to be elucidated. Further studies are necessary.

Anesthetic management of patients with M-A PAH needs to be performed more carefully than of those with idiopathic PAH patients, because M-A PAH patients show higher right atrial pressure, lower cardiac index and excise tolerance, and more dysfunctional RV than patients with idiopathic PAH [10]. Furthermore, a previous study reported that the 10-year prognosis of patients with M-A PAH was significantly poorer than those with idiopathic PAH (25% vs. 45.7%, respectively) [8]. We performed transversus abdominis plane block for postoperative analgesia and replaced the tracheal tube with a supraglottic airway after surgery in order to reduce noxious stimulation and prevent reflection to cough reflex or airway stimulation leading to worsening pulmonary hypertension. Peritoneal insufflation may deteriorate pulmonary hypertension by inducing hypercapnia. Although we administered nitroglycerin and dobutamine for controlling PAP, conversion from laparoscopic to open surgery might have been required. Judicious selection of anesthetic induction and maintenance agents is crucial in patients with PAH. There were two reasons why we selected thiopental as an induction agent in the present case. First, thiopental has the advantage of less impact on RV contractility and systemic vascular resistance compared to propofol. Second, the patient had been taking various antipsychotic medicines including benzodiazepines and antidepressants for sleep impairment and may have developed tolerance to benzodiazepine. Whereas propofol and volatile anesthesia markedly decrease RV contractility, opioid has minimal effects on pulmonary hemodynamics [4]. Therefore, we selected balanced anesthesia maintenance with sevoflurane and opioid. The agent to use as medication support for patients with PAH is still controversial. A previous study reported that noradrenaline and vasopressin are preferable to phenylephrine [11]. Furthermore, nitric oxide may be useful, as it reduces PVR without decreased systemic vascular resistance. In the present case, we did not use nitric oxide because its usage in patients undergoing non-cardiac surgery is not accepted under the Japanese health insurance system. In addition, patients that abuse methamphetamine have altered anesthetic requirements [12]; thus, it is necessary to use a BIS monitor to monitor the depth of anesthesia.

We monitored PAP using a pulmonary artery catheter, although its intraoperative use remains controversial [13]. We considered continuous monitoring of the mean PAP, systemic resistance, and cardiac index valuable for controlling the volume and administration of vasodilator and inotropic medication. Moreover, during peritoneal insufflation, the mean PAP encouraged the administration of a vasodilator.

Patients with long-term exposure to methamphetamine have high risks during the intraoperative period due to PAH. We successfully anesthetized a patient with M-A PAH by monitoring RV function and avoided increased pulmonary arterial resistance with appropriate anesthesia and medical hemodynamic support.

## Data Availability

The datasets used and/or analyzed during the present study are available upon reasonable request.

## Abbreviations

M-A PAH: Methamphetamine-associated pulmonary arterial hypertension
PVR: Pulmonary vascular resistance
RV: Right ventricular
PAP: Pulmonary arterial pressure
BIS: Bispectral index
TEE: Transesophageal echocardiography
